# Clinical Applications of CDK4/6 Inhibitors in HR+/HER2, and Personalized Treatment Strategies: A Narrative Review

**DOI:** 10.32604/or.2026.076300

**Published:** 2026-05-21

**Authors:** Guoliang Zhong, Tianqing Yang, Shuqi Lin, Muyi Zhong

**Affiliations:** 1 The First School of Clinical Medicine, Guangdong Medical University, Zhanjiang, China; 2 Department of Breast Surgery, Dongguan People’s Hospital, Dongguan, China

**Keywords:** Cyclin-dependent kinases 4 and 6 inhibitors, breast cancer, endocrine therapy, personalized treatment

## Abstract

Hormone Receptor-positive/Human Epidermal Growth Factor Receptor 2-negative (HR+/HER2−) breast cancer treatment has made a breakthrough due to the introduction of cyclin-dependent kinases 4 and 6 (CDK4/6) inhibitors. This article mainly reviews the mechanisms of action, clinical efficacy, and current application status of CDK4/6 inhibitors, including Palbociclib, Ribociclib, Abemaciclib, and the emerging Dalpiciclib. The advantages and limitations of different treatment stages are also discussed. CDK4/6 inhibitors have excellent efficacy in prolonging progression-free survival (PFS) and overall survival (OS), and have become a key option for HR+/HER2− breast cancer first-line and adjuvant treatment. The issues of drug resistance and adverse reactions remain severe. Strategies such as combining phosphoinositide 3-kinase (PI3K) inhibitors, immunotherapy, or new estrogen receptor (ER)-degrading agents are being actively explored for drug-resistant patients. The personalized precision therapy may become the core direction for optimizing the application of CDK4/6 inhibitors in the future, which will improve patients’ quality of life.

## 1 Introduction

**Figure 1:**
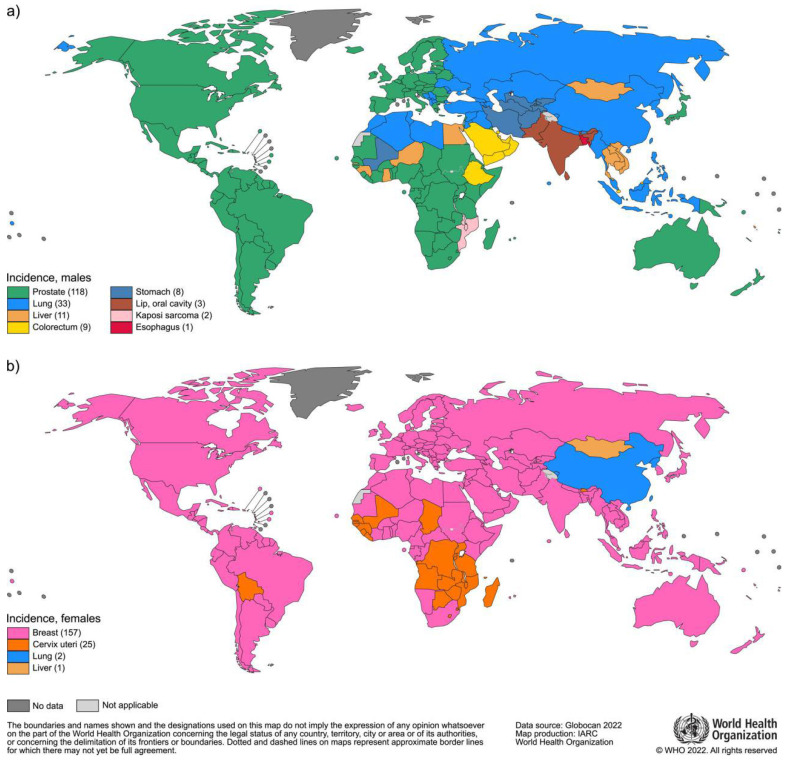
Global maps present the most common type of cancer incidence in 2022 in each country among (**a**) men and (**b**) women. The number of countries represented in each ranking group is included in the legend. Reproduced with permission. Copyright 2024, Journal of CA Cancer J Clin [1].

Based on data from GLOBOCAN 2022, approximately 2.30 million new cases of breast cancer were reported worldwide in 2022, representing 11.6% of all new cancer cases [1] (Fig. 1). According to the latest global cancer data, breast cancer has emerged as the leading cause of cancer-related death among women, with specific figures indicating that it is responsible for a significant number of fatalities annually [1].

Regarding hormone receptor (HR) testing, results for estrogen receptor (ER), progesterone receptor (PR), and human epidermal growth factor receptor 2 (HER2) can be positive or negative. Female breast cancer is broadly classified into four major molecular subtypes based on receptor status: HR+/HER2−, HR−/HER2−, HR+/HER2+, and HR−/HER2+ [2]. Among these, HR+/HER2− breast cancer is the most common subtype worldwide [3].

For patients with inoperable locally advanced breast cancer, neoadjuvant chemotherapy (NACT) is a primary treatment modality [4]. Conventionally, neoadjuvant chemotherapy (NAC) regimens were predominantly based on cytotoxic chemotherapeutic drugs. However, its scope has evolved considerably with advances in molecular oncology. The term now frequently encompasses, and is often integrated with, other systemic modalities such as targeted therapies (e.g., trastuzumab and pertuzumab for HER2-positive tumors) and immune checkpoint inhibitors (e.g., programmed death-1/programmed death-ligand 1 (PD-1/PD-L1) inhibitors), collectively termed “neoadjuvant systemic therapy” or simply “neoadjuvant therapy.” This therapy effectively reduces tumor volume preoperatively, decreases invasiveness, and can even lead to tumor downstaging, ultimately creating an opportunity for surgery. In patients planned for breast-conserving surgery, NACT increases the likelihood of preserving breast tissue by reducing tumor size, thereby enhancing quality of life and psychological satisfaction. However, one study indicated that patients with the specific and most common HR+/HER2− subtype achieve relatively poor rates of pathological complete response (pCR) following NACT. This suggests that even following NACT, tumors may not entirely vanish in some patients [4]. Moreover, NACT may induce a variety of side effects, including neutropenia, rash, alopecia, myelosuppression, nausea, and vomiting, all of which can substantially affect patients’ quality of life. Some patients might also acquire resistance to chemotherapeutic agents, thereby influencing treatment efficacy [4].

For patients with HR+/HER2− breast cancer, endocrine therapy (ET) is often the preferred option. Tumor cells in this subtype typically exhibit high expression of ER and/or PR, while HER2 receptor is usually not expressed or expressed at low levels. By inhibiting or blocking hormone action, endocrine therapy can slow or halt the growth of cancer cells, serving as a cornerstone in the treatment of HR+/HER2− breast cancer [5]. A study by Hilbers et al. suggested that patients with small, ER+/HER2− breast cancer and low genomic risk did not derive significant benefit from chemotherapy; those receiving endocrine therapy achieved an 8-year distant metastasis-free survival (DMFS) of 89.3%, compared to 79.4% in untreated patients [6]. This indicates that for HR+/HER2− breast cancer patients, endocrine therapy is generally superior to chemotherapy. A study by Liu et al. showed that endocrine therapy was superior to chemotherapy in terms of progression-free survival (PFS) and overall survival (OS), regardless of whether used as first-line, maintenance, or second-line therapy [7].

Regarding endocrine therapy, a Chinese society of clinical oncology (CSCO) study reported that among all local HR+/HER2− patients, the usage rate of CDK4/6 inhibitors combined with endocrine therapy accounted for 47.2% [8]. For the treatment of HR+/HER2− advanced or metastatic breast cancer (ABC/mBC), CDK4/6 inhibitors such as palbociclib, ribociclib, and abemaciclib have been approved by the US Food and Drug Administration (FDA) and the European Medicines Agency (EMA). These agents have demonstrated significant efficacy in both first-line and subsequent lines of therapy [9].

## 2 Literature Search Strategy

This narrative review was conducted following a comprehensive literature search to identify relevant clinical trials, real-world studies, and key reviews on the use of CDK4/6 inhibitors in HR+/HER2− breast cancer. It is important to note that this is a narrative synthesis of the literature, and no formal systematic review or meta-analysis was performed.

We searched PubMed, Embase, ClinicalTrials.gov, and the Cochrane Library for publications from January 2015 to November 2025. The search strategy combined terms and keywords including “CDK4/6 inhibitor”, “palbociclib”, “ribociclib”, “abemaciclib”, “dalpiciclib”, “HR+/HER2− breast cancer”, “hormone receptor positive HER2 negative breast cancer”, “advanced breast cancer”, “metastatic breast cancer”, “clinical trial”, “real-world evidence”, “biomarker”, and “resistance mechanism”.

The search was focused on phase II/III randomized controlled trials, large-scale real-world studies, key review articles, and meta-analyses that reported efficacy outcomes (such as progression-free survival and overall survival) or safety profiles. Publications were limited to those in English. Case reports, editorials, and conference abstracts without a peer-reviewed full text were not included, nor were studies not specifically focused on HR+/HER2− breast cancer.

Following the search, two authors independently screened the titles and abstracts, and subsequently reviewed the full texts of potentially relevant articles. Any discrepancies were resolved through discussion. Data on study design, patient characteristics, treatment regimens, efficacy outcomes, safety profiles, and biomarker correlates were extracted and synthesized narratively, with an emphasis on deriving comparative insights and discussing clinical implications.

## 3 Mechanism of Action of CDK4/6 Inhibitors

### 3.1 Cell Cycle Inhibition and G1 Phase Arrest

CDK4/6 inhibitors exert their antiproliferative effects by inhibiting Cyclin D/CDK4/6 complex activity, thereby blocking retinoblastoma protein (Rb) phosphorylation. This results in tumor cells being arrested in the G1 phase, preventing their progression to the S phase. This mechanism of action is particularly effective in ER-positive breast cancer cells, as these cancers generally retain a functional and intact Rb protein. Through this process, CDK4/6 inhibitors can significantly delay tumor cell growth [10,11].

### 3.2 Senescence Effect and Inhibition of Cell Proliferation

Beyond inhibiting cell cycle progression, CDK4/6 inhibitors can induce the formation of a senescent phenotype, accelerating irreversible arrest in tumor cells. The development of this senescent phenotype is typically associated with the upregulation of genes related to senescence, such as CDKN2A and CDKN1A, leading to irreversible proliferative arrest [11,12,13]. Notably, unlike traditional senescence models, senescence driven by palbociclib is characterized by reduced mTORC1 (mechanistic target of rapamycin complex 1) activity and upregulated autophagy. One study indicated that inhibition of mTORC1 activity can exacerbate the senescent phenotype; conversely, when mTORC1 is inhibited and autophagy is suppressed, senescence can be reversed. This suggests that autophagy plays a crucial role in maintaining the senescent state [14]. These diverse cell fate determination mechanisms enhance the antitumor efficacy of CDK4/6 inhibitors and provide multiple avenues for overcoming tumor resistance.

### 3.3 Immunomodulation of the Tumor Microenvironment

CDK4/6 inhibitors augment the antigen presentation capability of tumor cells and trigger the expression of endogenous retroviral elements (ERVs). This promotes the production of type III interferon and strengthens the immune system’s ability to recognize tumors. A specific manifestation is the increased expression of MHC-I molecules on the tumor cell surface, which makes the tumor cells more recognizable to T cells of the immune system [12,15]. Moreover, they can selectively inhibit the proliferation of regulatory T cells (Tregs) while not affecting effector T cells, thereby helping to restore antitumor immune activity.

### 3.4 Metabolic Effects and Tumor Cell Adaptation

**Figure 2:**
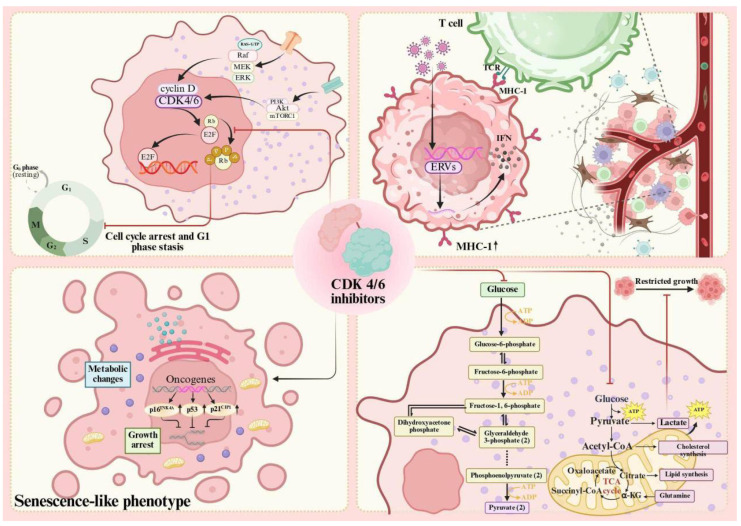
Mechanism of Action of CDK4/6 Inhibitors. Note: RAS-GTP, GTP-bound RAS protein; RAF, Raf proto-oncogene, serine/threonine kinase; MEK, mitogen-activated protein kinase kinase; ERK, extracellular signal-regulated kinase; PI3K, phosphoinositide 3-kinase; Akt, protein kinase B; mTORC1, mechanistic target of rapamycin complex 1; ERVs, endogenous retroviruses; MHC-1, major histocompatibility complex class I; IFN, interferon; TCR, T cell receptor. Created in BioRender, Zhong (2025) [10,11,12,14,15].

The role of CDK4/6 inhibitors in cellular metabolism is noteworthy, as it inhibits glycolysis and mitochondrial oxidative metabolism, consequently impacting the energy supply of tumor cells. By inhibiting these metabolic pathways, the energy necessary for tumor cell growth and division is restricted, thereby further suppressing tumor cell proliferation [15]. This metabolic interference not only directly inhibits the proliferative capacity of tumor cells but may also enhance their sensitivity to other treatments, thereby laying a theoretical foundation for combination strategies [11] (Fig. 2).

## 4 Clinical Application of CDK4/6 Inhibitors in Advanced Breast Cancer

### 4.1 Importance of CDK4/6 Inhibitors in Advanced Breast Cancer Treatment

Literature suggests [16] that the combination of CDK4/6 inhibitors and endocrine therapy significantly improves progression-free survival (PFS) and overall survival (OS) in patients with HR+/HER2− breast cancer.

CDK4/6 inhibitors also exhibit unique and favorable safety profiles compared to traditional regimens. For instance, CDK4/6 inhibitor-associated neutropenia is characterized by rapid reversibility, reflecting a transient cytostatic effect on myeloid neutrophil precursors, in contrast to the irreversible damage caused by chemotherapy. For example, in the PALOMA series trials, the incidence of neutropenia events in the palbociclib combination groups was 75–98%, but the incidence of febrile neutropenia was very low (<2%), significantly lower than with conventional chemotherapy [17]. Furthermore, the rate of adverse reactions necessitating treatment discontinuation is lower with CDK4/6 inhibitors than with chemotherapy, rendering them more tolerable for patients during treatment [17].

### 4.2 Rapid Development in Clinical Application for Advanced Breast Cancer

The combination of CDK4/6 inhibitors and endocrine therapy has brought about a transformative shift in the treatment landscape for HR+/HER2− metastatic breast cancer. Numerous phase III randomized studies and real-world evidence, such as the RIGHT Choice and RIBANNA studies, substantiate that the combination of CDK4/6 inhibitors with endocrine therapy significantly extends progression-free survival (PFS) and overall survival (OS) in patients with HR+/HER2− breast cancer, compared to endocrine monotherapy [18,19]. The PALOMA-2 study demonstrated that first-line use of palbociclib plus letrozole significantly extended the median PFS compared to letrozole alone, and the subsequent PALOMA-3 trial confirmed a similar benefit for palbociclib plus fulvestrant [20,21]. In the following years, phase III studies of abemaciclib and ribociclib reported encouraging results in patients with HR+/HER2− metastatic breast cancer [22]. A network meta-analysis indicated that all four CDK4/6 inhibitors (palbociclib, ribociclib, abemaciclib, dalpiciclib) combined with an aromatase inhibitor (AI) significantly improved PFS compared to AI alone, with hazard ratios ranging from 0.51 to 0.59. Among them, dalpiciclib plus AI ranked best for PFS (surface under the cumulative ranking analysis (SUCRA) value 77.9%), while abemaciclib plus AI ranked highest for objective response rate (ORR) and clinical benefit rate (CBR) (89.3% and 68.9%, respectively). These drugs demonstrated consistent treatment benefits across various patient subgroups, establishing CDK4/6 inhibitor plus endocrine therapy as the standard first-line treatment for HR+/HER2− advanced breast cancer and providing important evidence-based medicine for clinical decision-making [23].

In a series of clinical trials, not only the three approved CDK4/6 inhibitors but also newer agents like dalpiciclib have shown great promise. When used in combination with endocrine drugs like fulvestrant, dalpiciclib notably extended patients’ PFS [24].

Clinical research on new drugs within the CDK4/6 inhibitor class is providing a wider range of options. Dalpiciclib has demonstrated substantial efficacy in specific patient populations that have developed resistance to prior therapies. This further facilitates the expanding application of CDK4/6 inhibitors in breast cancer treatment [24].

Moreover, related drug resistance mechanisms and strategies to overcome them are being actively explored at the clinical level. Acquired resistance is rooted in a series of critical molecular lesions that collectively subvert drug-induced proliferative arrest. At the core of the cell cycle machinery, loss of RB1 function directly abolishes the G1 checkpoint, while amplification of Cyclin E1 establishes a fully independent proliferative engine via activation of CDK2; both represent the most direct mechanisms for bypassing CDK4/6 inhibition [25]. Furthermore, loss-of-function mutations in the FAT1 gene lead to CDK6 overexpression and the formation of resistant CDK6-INK4 complexes via the Hippo pathway, constituting another key genetic resistance route. Rewiring of upstream signaling pathways is crucial: PIK3CA mutations, by constitutively activating the PI3K-AKT-mTOR axis, not only upregulate Cyclin D but also sustain pro-survival signals through complex feedback loops. Concurrently, ESR1 mutations confer ligand-independent, constitutive activation of the estrogen receptor, rendering tumor cells completely insensitive to the endocrine therapy component of combination regimens and thereby dismantling the therapeutic synergy. These alterations in upstream pathways often converge on the upregulation of the global transcriptional amplifier c-Myc, whose expression levels inversely correlate with the efficacy of CDK4/6 inhibitors, making it a core driver of resistance [25]. The nodal regulation of cell fate is equally critical: wild-type TP53 function is essential for facilitating the CDK4/6 inhibitor-induced senescence-like phenotype, whereas TP53 mutation impedes entry into stable growth arrest, trapping cells in a precarious equilibrium state more amenable to adaptation and escape, thereby paving the way for the development of other resistance mechanisms [26]. Notably, approximately 70% of resistant cases show no new somatic mutations, highlighting the importance of non-genetic mechanisms, including c-Myc-driven low-level E2F activation, AMBRA1 loss leading to Cyclin D-CDK2 complex formation, AURKA amplification, and adaptive changes involving specific miRNAs (e.g., miR-432-5p) and epigenetic remodeling (e.g., enhanced AP-1 activity). Immune modulatory changes in the tumor microenvironment, such as Treg cell suppression and altered MHC molecule expression, also contribute to the resistance process [25]. These mechanisms do not operate in a linear, additive manner but are dynamically combined during tumor clonal evolution. For instance, a clone harboring both a PIK3CA mutation (providing a survival advantage) and a TP53 mutation (enabling escape from senescence) may subsequently acquire Cyclin E1 amplification or sustained c-Myc activation, ultimately evolving a fully resistant phenotype [25,26].

In response to these multi-layered and dynamically evolving resistance mechanisms, current research is focused on developing tiered and synergistic counter-strategies. To target the core cell cycle bypass activation, the combination or sequential use of CDK2 inhibitors has become a central approach to overcome resistance driven by Cyclin E1 amplification or TP53 loss. To address the rewiring of upstream driver signals, strategies involve combining PI3K/AKT/mTOR pathway inhibitors (e.g., Alpelisib, Capivasertib) with CDK4/6 inhibitors or exploring CDK7 inhibitors (e.g., Samuracidib), aiming to block escape routes at the level of transcription and signaling nodes [27]. Regarding clinical decision-making after disease progression, options include switching to a different class of CDK4/6 inhibitor while maintaining endocrine therapy, leveraging their potentially distinct inhibitory profiles. Furthermore, building on the immunomodulatory potential of CDK4/6 inhibitors, their combination with immune checkpoint inhibitors is under investigation, although this strategy currently faces clinical challenges of uncertain efficacy and overlapping toxicities. These diversified, mechanism-informed strategies are systematically expanding the application dimensions and future potential of CDK4/6 inhibitors in clinical practice [27].

## 5 Current Status of Clinical Research on CDK4/6 Inhibitors

### 5.1 Abemaciclib

Clinical trials such as MONARCH 2 have demonstrated that Abemaciclib significantly extends progression-free survival (PFS) and improves objective response rates (ORR) in the first-line treatment of hormone receptor-positive (HR+), human epidermal growth factor receptor 2-negative (HER2−) advanced breast cancer [28]. The MONARCH 3 trial evaluated the efficacy of abemaciclib combined with a non-steroidal aromatase inhibitor (NSAI) as first-line therapy for HR+/HER2− advanced breast cancer (ABC). Results showed that the median PFS in the abemaciclib group was 29.0 months, significantly superior to 14.8 months in the placebo group (HR = 0.535, *p* < 0.0001). Chemotherapy-free survival (CFS) was also extended from 30.6 months in the control group to 46.7 months (HR = 0.693, *p* = 0.0010), indicating its effectiveness in delaying the need for more aggressive therapy. Although OS was prolonged by 13.1 months (66.8 vs. 53.7 months), statistical significance was not reached (HR = 0.804, *p* = 0.0664). However, subgroup analysis revealed that patients with visceral metastases exhibited a more significant OS advantage (63.7 vs. 48.8 months), suggesting a potentially larger therapeutic value for this subgroup. Regarding safety, the incidence of grade ≥ 3 adverse events was relatively high, including neutropenia (27.5%), diarrhea (9.8%), venous thromboembolism (7.6%), and interstitial lung disease (7.0%), but overall patient tolerance was good. In summary, abemaciclib combined with NSAI significantly improves PFS and CFS in patients with HR+/HER2− ABC, particularly showing potential survival benefit for those with visceral metastases, supporting its selection as first-line therapy for corresponding patients [29].

In the adjuvant treatment of patients with HR+/HER2− early breast cancer at high risk of recurrence, the monarchE trial confirmed that abemaciclib combined with endocrine therapy (ET) significantly reduces the risk of disease recurrence and provides long-term clinical benefit. Data showed that the 4-year invasive disease-free survival (iDFS) rate was 85.8% in the abemaciclib group, significantly better than 79.4% in the ET-alone group (HR = 0.664, 95% CI: 0.578–0.762, *p* < 0.0001). Distant relapse-free survival (DRFS) was also significantly improved (88.4% vs. 82.5%, HR = 0.659, *p* < 0.0001), demonstrating its effectiveness in suppressing distant metastasis [30].

Study follow-up data revealed that the iDFS benefit increased over time (absolute improvement of 2.8% at 2 years, 4.8% at 3 years, and 6.4% at 4 years). This suggests that abemaciclib may possess a persistent “carry-over effect”, meaning patients continue to derive benefit even after treatment completion [30]. A subsequent 5-year follow-up analysis confirmed this observation. In that analysis, the absolute iDFS and DRFS benefits at 5 years reached 7.6% and 6.7%, respectively, significantly higher than the corresponding 4.8% and 4.1% at 3 years, clearly indicating sustained benefit. Although OS did not reach statistical significance (HR = 0.903, *p* = 0.284), fewer death events occurred in the abemaciclib group (208 vs. 234), hinting at an emerging survival benefit [31]. Regarding safety, the main adverse reactions included grade 3-4 neutropenia (19.6%), diarrhea (7.8%), venous thromboembolism (VTE, 2.5%), and interstitial lung disease (ILD, 7.0%). However, overall patient tolerance was good, with no new significant safety signals identified [30]. The monarchE trial follow-up data established the role of adjuvant abemaciclib in patients with high-risk early HR+/HER2− breast cancer. Abemaciclib is particularly effective in reducing distant recurrence and continuously improving iDFS, offering a new treatment option for this patient population and further highlighting the value of CDK4/6 inhibitors in early breast cancer [30].

A real-world study, for the first time, delineated the clinical practice patterns of adjuvant abemaciclib use in Japanese patients with HR+/HER2− early breast cancer. Based on the Medical Data Vision database, this study included 374 early breast cancer patients receiving adjuvant abemaciclib between December 2021 and March 2023. The median age was 54 years (IQR: 46–65), and 99.2% were female. Treatment patterns showed that 38.2% of patients received neoadjuvant chemotherapy, 51.6% received adjuvant chemotherapy, and 63.4% received radiotherapy. Notably, 13.1% of patients did not receive any perioperative chemotherapy prior to abemaciclib. Regarding safety, 42.0% of patients required dose reduction (≥50 mg), with higher rates observed in elderly patients (≥65 years, 46.81%) and those with low BMI (<18.5 kg/m^2^, 52.94%), however, consistent treatment benefits were observed across all age subgroups. Supportive care measures, including antidiarrheals and probiotics, were administered to 95.19% of patients, indicating diarrhea as the predominant adverse reaction to abemaciclib. This study demonstrated that adjuvant abemaciclib treatment patterns in Japanese clinical practice align closely with those observed in the MonarchE trial, offering critical real-world evidence for its application in Asian populations [32].

### 5.2 Palbociclib

In the treatment of advanced HR+/HER2− breast cancer, the combination of palbociclib—a CDK4/6 inhibitor—and endocrine therapy has become a standard regimen. Its clinical efficacy was confirmed in the PALOMA-1 study. Results demonstrated that treatment with palbociclib and the non-steroidal aromatase inhibitor letrozole significantly extended median PFS to 20.2 months, nearly double the 10.2 months observed with letrozole monotherapy (HR = 0.488, 95% CI 0.319‒0.748, *p* = 0.0004). This indicates that palbociclib effectively delays disease progression. In the palbociclib group, the time from randomization to the first chemotherapy was 26.7 months, which was significantly later than the 17.7 months in the letrozole group. This suggests clinical value in delaying the need for more aggressive therapy [33].

Regarding overall survival (OS), the median OS in the palbociclib group was 37.5 months, which was longer than the 34.5 months in the letrozole group. However, this difference was not statistically significant (HR = 0.897, 95% CI 0.623–1.294, *p* = 0.281). From a safety perspective, adverse reactions in the palbociclib group predominantly manifested as hematologic toxicities, with a 59% incidence of grade 3-4 neutropenia. Notably, no severe febrile neutropenia was observed, suggesting that the toxicity was relatively manageable. The use of palbociclib significantly improves PFS, delays disease progression, and has an acceptable safety profile, providing a safe and effective treatment option for patients with advanced HR+/HER2− breast cancer [33].

In a Japanese real-world evidence (RWE) study involving 677 patients, 420 received palbociclib as first-line therapy, and 257 received it as second-line therapy. The data showed that the median PFS in the first-line group was 24.5 months (95% CI 19.9–29.4), demonstrating an advantage over the second-line group of 14.5 months (95% CI 10.2–19.0), further validating the robust efficacy of palbociclib in the first-line setting. With regard to safety, the dose adjustment rate in this study was relatively high, reaching 73.6% [34]. Nevertheless, dose reduction did not affect treatment efficacy, aligning with findings from prior PALOMA studies. In the real-world setting, the objective response rate (rwORR) was 37.9% for first-line treatment and 23.0% for second-line treatment, while the clinical benefit rate (rwCBR) reached 76.7% and 66.9%, respectively. These findings further underscore the clinical value of palbociclib in managing HR+/HER2− advanced breast cancer (ABC) patients [34].

The Young-PEARL trial was a randomized, open-label, phase II study conducted in South Korea, designed to compare palbociclib plus exemestane with ovarian function suppression versus capecitabine monotherapy in premenopausal women with HR+/HER2− metastatic breast cancer who had progressed following prior tamoxifen therapy. With an extended median follow-up of 54.0 months (IQR 34.1–74.4), the final overall survival analysis demonstrated that while the palbociclib-based combination continued to show a significant progression-free survival benefit (median PFS 19.5 months vs. 14.0 months; hazard ratio 0.74, 90% CI 0.57–0.98), there was no statistically significant difference in median overall survival between the two groups (palbociclib group 54.8 months, 95% CI 48.9–77.1; capecitabine group 57.8 months, 95% CI 46.3–89.2; hazard ratio 1.02, 95% CI 0.69–1.51; *p* = 0.92). Notably, 42% of patients in the capecitabine arm received a CDK4/6 inhibitor as subsequent therapy after the study, and multivariable analysis identified the use of a CDK4/6 inhibitor after disease progression as the only factor significantly associated with longer overall survival. Regarding safety, the most common grade ≥ 3 adverse event in the palbociclib arm was neutropenia (64% vs. 18% in the capecitabine arm), aligning with the known profile of the regimen. These findings suggest that for premenopausal patients, a CDK4/6 inhibitor-based combination remains the preferred first-line option due to its clear PFS advantage and improved quality of life [35].

The unique advantages of palbociclib in the elderly patient population should not be overlooked. Studies of the PALOMA series and 26 real-world datasets suggest that elderly patients, particularly those ≥ 75 years old, can achieve comparable progression-free survival (PFS) benefits from targeted therapies such as palbociclib and pembrolizumab, as observed in the PALOMA-3 study and other real-world evidence. Regarding adverse events, the incidence of grade 3-4 neutropenia in the elderly group was high (46.4%–63%), but the risk of infection did not increase significantly, suggesting good tolerability in elderly patients. Palbociclib had no significant impact on the quality of life (QoL) of elderly patients, as evidenced by most patients maintaining good functional status after 6 months of treatment. Palbociclib exhibits stable efficacy and manageable safety in treating elderly HR+/HER2− advanced breast cancer patients, underscoring its importance as a treatment option for this group. Individualized dose adjustments made according to patient tolerance can improve treatment compliance and efficacy [36].

The value of palbociclib in the adjuvant setting for early breast cancer remains unestablished. The phase III PALLAS trial showed that the addition of palbociclib to adjuvant endocrine therapy did not improve iDFS (4-year rate 84.2% vs. 84.5%, HR = 0.96, *p* = 0.65), with no significant improvement in DRFS or OS either. Notably, 44.9% of patients failed to complete the planned 2-year treatment course due to adverse reactions like neutropenia, which indicates challenges in tolerability for early breast cancer patients [37]. The PENELOPE-B trial yielded similar results: palbociclib failed to demonstrate clinical benefit in high-risk patients with residual disease after neoadjuvant chemotherapy. This stands in contrast to the positive results of abemaciclib in the monarchE trial, suggesting potential differences in the mechanisms of action or suitable patient populations among different CDK4/6 inhibitors [38].

### 5.3 Ribociclib

Ribociclib has demonstrated significant survival benefits in the first-line treatment of premenopausal and perimenopausal women with HR+/HER2− advanced breast cancer (ABC). According to the MONALEESA-7 trial, with 53.5 months of follow-up data, the median overall survival (OS) for patients treated with ribociclib in combination with endocrine therapy (ET) was 58.7 months, significantly surpassing the 48.0 months observed in the control group (HR = 0.76, *p* < 0.001). This translates to a 4-year survival rate of 60% compared to 50% in the control group. Regarding disease control, the median PFS in the combination group was 23.8 months, nearly double that of ET alone (13.0 months) (HR = 0.55, *p* < 0.001), and significantly reduced the risk of distant metastasis (43% vs. 52%). Notably, ribociclib significantly delayed the time to first chemotherapy (50.9 vs. 36.8 months, HR = 0.69) and prolonged chemotherapy-free survival (42.4 vs. 26.4 months, HR = 0.67), while maintaining patient quality of life. The safety profile was consistent with previous studies, with grade ≥ 3 neutropenia (65%) being the main adverse event, but no new safety signals emerged, and overall tolerability was manageable. This study established the position of ribociclib as a first-line treatment in this population, highlighting the core value of CDK4/6 inhibitors in the comprehensive management of advanced breast cancer [39].

For the adjuvant treatment of stage II or III early HR+/HER2− breast cancer, an interim analysis of the NATALEE trial confirmed that ribociclib combined with an NSAI can significantly reduce the risk of recurrence and may enhance control over distant metastasis. Data showed that the 3-year iDFS rate in the ribociclib group was 90.4%, significantly superior to 87.1% in the NSAI-alone group (HR = 0.75, *p* = 0.003). The significant improvement in distant disease-free survival (DDFS) to 90.8% compared to 88.6% (HR = 0.74) underscores its efficacy in preventing distant metastasis, as supported by clinical trials. Although the current OS data are not yet mature (HR = 0.76, *p* = 0.54), the mortality rate was lower in the Compared with the control group, the ribociclib group showed certain differences, but further follow-up is needed to obtain evidence for a long-term survival advantage. As for safety, the main adverse reactions comprised grade ≥ 3 neutropenia (43.8%), liver function abnormalities (8.9%), and QT interval prolongation (5.2%). Nevertheless, overall patient tolerance proved to be good, and no new safety signals emerged. The 3-year follow-up data from the NATALEE trial established the role of adjuvant ribociclib in patients with HR+/HER2− early breast cancer. It is particularly remarkable in reducing distant recurrence and consistently enhancing iDFS. This provides a new treatment option for such patients and further supports the value of CDK4/6 inhibitors in early breast cancer [40].

### 5.4 Others (Dalpiciclib/Trilaciclib)

Dalpiciclib, a new-generation CDK4/6 inhibitor, has exhibited outstanding efficacy and safety in treating HR+/HER2− advanced breast cancer, with distinct advantages in the Chinese population. Based on the DAWNA-1 study (NCT03481998), the combination of dalpiciclib and fulvestrant significantly improved PFS in patients who had progressed on prior endocrine therapy, with a median PFS of 15.7 months (HR = 0.424, *p* < 0.0001), more than double that of fulvestrant alone (7.2 months) [41]. Furthermore, in the DAWNA-2 study (NCT03966898), the median PFS for dalpiciclib combined with an AI in treatment-naïve HR+/HER2− ABC patients was as high as 30.6 months, with an ORR of 67.6%, showing efficacy comparable to ribociclib and abemaciclib. The safety profile of dalpiciclib is characterized by reversible hematologic toxicity, with grade 3-4 neutropenia incidence of 61.8%–87.5%, but no febrile neutropenia (FN) was reported. Gastrointestinal adverse reactions are relatively mild, suggesting better patient tolerability. Additionally, its unique pharmacokinetic properties, with a half-life ranging from 48.0 to 53.6 h, may contribute to stable efficacy and improved patient compliance [42]. With the advancement of phase III trials, the CSCO guidelines have recommended the inclusion of dalpiciclib for the treatment of HR+/HER2− breast cancer patients, and it is expected to expand into the adjuvant setting for high-risk early breast cancer patients, establishing it as a representative domestic CDK4/6 inhibitor in China and further promoting the development of personalized breast cancer treatment [43].

Trilaciclib, a novel intravenously administered CDK4/6 inhibitor, has demonstrated notable clinical benefits in the treatment of metastatic triple-negative breast cancer (mTNBC). This drug can transiently arrest hematopoietic stem/progenitor cells and immune cells in the G1 phase, thereby shielding these cells from chemotherapy-induced damage during treatment and potentially enhancing immune activity [44]. In a randomized phase II study, administering trilaciclib prior to gemcitabine plus carboplatin (GCb) chemotherapy significantly improved OS compared to GCb chemotherapy alone, with median OS extending from 12.6 months to 19.8 months (HR = 0.37; *p* < 0.0001). Importantly, the antitumor efficacy enhancement effect of trilaciclib was independent of the tumor’s CDK4/6 dependency status, showing similar efficacy in CDK4/6-dependent, independent, or indeterminate tumors. Furthermore, although a greater survival benefit was observed in PD-L1-positive patients, trilaciclib prolonged OS regardless of PD-L1 expression status. According to T-cell receptor analysis, the application of trilaciclib enhanced T-cell activation and Trilaciclib has been shown to enhance the activation of antitumor T cells and alter the tumor microenvironment, suggesting its potential to exert antitumor effects through immunomodulatory mechanisms. These findings indicate that trilaciclib can enhance the efficacy of chemotherapy and combination therapies through multiple mechanisms, offering an innovative strategy for mTNBC patients that both protects normal cells and enhances antitumor immune responses [44] (Table 1).

**Table 1:** Key features comparison of major CDK4/6 inhibitors in HR+/HER2− breast cancer.

CDK4/6 Inhibitor	Key Research	Progression-Free Survival (mPFS) in Late-Stage Patients	OS in Advanced Patients	Early Patient	Main Hematological Toxicity	Unique Advantages	Clinical Considerations
**Abemaciclib**	MONARCH 3, monarchE	29.0	Not statistically significant	5-year IDFS and DRFS showed significant improvement	Intermediate to low (neutropenia grade 3-4: ~20%)	Potential advantages for patients with advanced visceral metastases and early high recurrence risk; strong evidence for adjuvant therapy	Preventive treatment for diarrhea is required; pay attention to the risk of VTE/ILD
**Palbociclib**	PALOMA-2, PALOMA-3	20.2	Not statistically significant	Neither IDFS, DRFS, nor the OS showed significant improvements	High (neutropenia grade 3-4: ~60%)	The elderly patients had good tolerance	Suitable for patients with bone marrow suppression
**Ribociclib**	MONALEESA-7, NATALEE	23.8 (premenopausal)	Significant difference (premenopausal: 58.7 vs. 48.0 months)	After 3 years, IDFS and DDFS showed significant improvements, while OS data remains immature	High (neutropenia grade 3-4: ~65%)	OS benefits are well established in premenopausal/peri-menopausal patients	Caution is required in patients with underlying heart disease
**Dalpiciclib**	DAWNA-1, DAWNA-2	30.6	-	-	High (neutropenia grade 3-4: ~61–87.5%)	Gastrointestinal reactions are mild	China has abundant population data; safety features are distinctive

Note: OS, Overall Survival; IDFS, Invasive Disease-Free Survival; DDFS, Distant Disease-Free Survival; DRFS, Distant Recurrence-Free Survival. “-” indicates that the corresponding data were not reported in the cited studies or were not within the scope of the research.

## 6 Comparative Analysis

### 6.1 Differential Efficacy Profiles

Current clinical evidence indicates that while all CDK4/6 inhibitors combined with endocrine therapy significantly prolong progression-free survival (PFS), they differ in terms of overall survival (OS) benefit, preferential patient subgroups, and efficacy against specific metastatic sites. Ribociclib has demonstrated a clear OS benefit in several first-line studies (e.g., MONALEESA-2, -7) [45,46], particularly in pre-/perimenopausal patients, establishing it as a preferred option for this group and for those with *de novo* advanced disease or a longer disease-free interval (≥12 months). Abemaciclib, due to its higher selectivity for CDK4 [45,46], shows a pronounced PFS advantage in patients with visceral metastases or endocrine resistance (MONARCH-3), and its higher lipophilicity suggests potential utility in patients with brain metastases [46]. Palbociclib did not achieve a statistically significant OS benefit in first-line settings; its core value lies in substantially extending PFS and delaying the need for chemotherapy, with its tolerability validated in elderly patients [45,46,47]. The emerging agent dalpiciclib has shown comparable PFS benefit with a distinct pharmacokinetic profile in Chinese population data, providing an important option for these patients [43]. A recent large-scale real-world study (PALMARES-2) has further confirmed and refined these differences: in the overall population, both abemaciclib and ribociclib were associated with superior real-world PFS compared to palbociclib; in patients with endocrine-sensitive disease, only abemaciclib outperformed palbociclib, whereas in those with endocrine-resistant, Luminal B-like, or premenopausal disease, both abemaciclib and ribociclib were more effective than palbociclib. Notably, in patients with *de novo* metastatic disease, abemaciclib showed superior efficacy compared to both ribociclib and palbociclib. Furthermore, the three inhibitors showed similar effectiveness in patients with bone-only disease, while in older patients, palbociclib was not inferior to ribociclib. This collective evidence underscores the importance of individualized selection of CDK4/6 inhibitors based on disease aggressiveness, endocrine sensitivity, metastatic patterns, and patient-specific factors such as age and comorbidities [48].

### 6.2 Contrasting Safety Profiles and Clinical Management Considerations

The distinct adverse event profiles of different inhibitors significantly impact treatment choice and patient management. The primary dose-limiting toxicities for palbociclib and ribociclib are hematologic (grade 3–4 neutropenia > 60%), but the risk of febrile neutropenia is low, typically manageable via dose adjustment or supportive care [45,46]. Ribociclib requires additional monitoring for QTc interval prolongation risk (~16% incidence), warranting caution in patients with a history of arrhythmia or those on concomitant QTc-prolonging medications [45,46,47]. Abemaciclib causes relatively milder myelosuppression (grade 3-4 neutropenia ~22.5%) but is associated with prominent gastrointestinal toxicity, especially diarrhea (>85% incidence), often necessitating prophylactic management and early intervention [45,46]. Dalpiciclib’s toxicity profile is similar to palbociclib, characterized mainly by reversible hematologic toxicity, with reportedly milder gastrointestinal reactions [42].

### 6.3 Applicability Considerations in Special Populations

Patient comorbidities and clinical characteristics directly influence drug selection. For elderly patients (≥65 years), hematologic toxicity rates are higher with palbociclib and ribociclib, frequently requiring dose adjustments, whereas dose modifications with abemaciclib are more often due to diarrhea. For premenopausal patients, ribociclib is the only CDK4/6 inhibitor that, combined with ovarian function suppression (OFS), has demonstrated a clear OS benefit in the first-line setting. In patients with significant comorbidities (e.g., poor bone marrow reserve), abemaciclib’s lower hematologic toxicity may be advantageous. For those with underlying cardiac conditions, cautious evaluation is needed before using ribociclib [45,46].

### 6.4 Post-Resistance Treatment Strategies

Management after progression on a CDK4/6 inhibitor is a clinical challenge. Abemaciclib demonstrates unique value in this setting, as it may retain efficacy following resistance to palbociclib due to distinct underlying mechanisms. Preclinical and clinical studies indicate that palbociclib-resistant tumors, often characterized by upregulation of G2/M pathways, remain sensitive to abemaciclib, with a reported median PFS of 6.1 months and a significant overall survival benefit when used sequentially [49,50]. The postMONARCH trial confirmed that after progression on first-line CDK4/6 inhibitor (mostly palbociclib or ribociclib) plus endocrine therapy, switching to fulvestrant while continuing abemaciclib still significantly improved PFS [49,50]. This provides an effective “continue CDK4/6 inhibition, switch endocrine backbone” strategy post-resistance, particularly for patients without actionable genomic alterations. Evidence for similar sequential strategies with other inhibitors is still accumulating.

## 7 Summary and Outlook

The treatment paradigm for HR+/HER2− breast cancer has been fundamentally reshaped by the integration of CDK4/6 inhibitors with endocrine therapy. These agents have delivered unprecedented improvements in progression-free survival (PFS) and, for some, overall survival (OS), establishing a new standard of care in both the advanced and, increasingly, the high-risk early-stage settings. However, the current clinical landscape is characterized not by a single solution but by multiple effective agents with distinct profiles. The central challenge now lies in strategically navigating these options and addressing the inevitable development of resistance to maximize and prolong clinical benefit for each individual patient [47,51].

Looking ahead, the trajectory of CDK4/6 inhibitor therapy will be defined by several key avenues of optimization. First, the move from a “one-size-fits-all” to a biomarker-guided, precision approach is paramount. Identifying robust predictive biomarkers—beyond hormone receptor status—is crucial to select patients most likely to derive profound and durable benefit from initial CDK4/6 inhibition and to guide subsequent therapy choices. Promising tools, such as gene expression signatures and molecular profiling for alterations in RB1, ESR1, PIK3CA, and cyclin E, are under active investigation to fulfill this need [52]. Integrating the findings from Luo et al., who identified a 13-gene signature (including TFF3 and MGP) from baseline tumor cells that predicts late progression, along with distinct immune microenvironment features such as increased CD8^+^ T and NK cell infiltration in responders, provides tangible examples of such predictive biomarkers already emerging from single-cell transcriptomic studies [53]. These insights reinforce the potential of biomarker-driven strategies to personalize CDK4/6 inhibitor therapy and improve outcomes in HR+/HER2− metastatic breast cancer.

Second, overcoming acquired resistance requires innovative combination strategies. The future lies in rationally pairing CDK4/6 inhibitors with targeted agents that address specific escape pathways. A prime example is the combination with inhibitors of the PI3K/AKT/mTOR pathway—a critical co-driver of resistance frequently dysregulated in breast cancer through PIK3CA mutations or PTEN loss. Agents targeting this axis, such as the PI3Kα inhibitor alpelisib (approved for PIK3CA-mutant HR+/HER2− disease), AKT inhibitors like capivasertib, or mTOR inhibitors like everolimus, are being clinically explored alongside CDK4/6 inhibitors to achieve deeper and more durable pathway suppression and counteract cross-talk-mediated resistance. The clinical validity of this approach is supported by the phase III INAVO120 trial [54], which demonstrated that in patients with PIK3CA-mutated disease who relapsed early during or after adjuvant endocrine therapy, the triplet combination of the PI3Kα inhibitor inavolisib plus palbociclib and fulvestrant significantly improved overall survival (median OS: 34.0 vs. 27.0 months) and progression-free survival (median PFS: 17.2 vs. 7.3 months), along with a substantially higher objective response rate (62.7% vs. 28.0%), compared with placebo plus palbociclib-fulvestrant. Beyond this, CDK4/6 inhibitors are also being combined with other targeted agents such as novel estrogen receptor degraders (SERD), as exemplified by the phase III SERENA-6 trial [55], which demonstrated that switching to the oral SERD camizestrant upon ctDNA-detected ESR1 mutation (while continuing a CDK4/6 inhibitor) significantly improved progression-free survival (median 16.0 vs. 9.2 months) and delayed quality-of-life deterioration compared to continuing an aromatase inhibitor-based regimen. Other strategies include combining CDK4/6 inhibitors with antibody-drug conjugates to tackle heterogeneous resistance mechanisms. Furthermore, leveraging the immunomodulatory effects of CDK4/6 inhibition through combinations with immunotherapy represents a promising frontier, particularly in reshaping the immunosuppressive tumor microenvironment—a strategy that aligns with similar efforts to combine PI3K/AKT/mTOR inhibitors with checkpoint blockers. Ultimately, the expansion of the therapeutic arsenal also includes the development of next-generation CDK4/6 inhibitors like dalpiciclib and supportive novel agents such as the myeloprotective trilaciclib, aiming to improve both efficacy and tolerability profiles within these combinatorial frameworks [56,57,58].

Finally, refining the application of these drugs across the disease continuum is essential. While their role in advanced cancer is solidified, optimizing their use in the adjuvant and neoadjuvant settings for early breast cancer requires further refinement to identify the specific patient subsets that will benefit most, balancing efficacy against long-term tolerability. The ultimate goal is to transform HR+/HER2− breast cancer into a chronic, manageable condition through sequential, intelligent, and personalized treatment strategies. The journey of CDK4/6 inhibitors, from revolutionary breakthrough to refined precision tool, continues to evolve, holding the promise of further improving long-term outcomes for patients worldwide.

## Data Availability

The data that support the findings of this study are available from the corresponding author upon reasonable request.
